# Advanced imaging use and delays among inpatients with psychiatric comorbidity

**DOI:** 10.1002/brb3.3425

**Published:** 2024-02-15

**Authors:** Emily Bartsch, Saeha Shin, Kathleen Sheehan, Michael Fralick, Amol Verma, Fahad Razak, Lauren Lapointe‐Shaw

**Affiliations:** ^1^ Division of General Internal Medicine University of Toronto Toronto Ontario Canada; ^2^ Li Ka Shing Knowledge Institute St. Michael's Hospital Toronto Ontario Canada; ^3^ Department of Psychiatry University of Toronto Toronto Ontario Canada; ^4^ Centre for Mental Health University Health Network Toronto Ontario Canada; ^5^ Division of General Internal Medicine Sinai Health Toronto Ontario Canada; ^6^ Division of General Internal Medicine Unity Health Toronto Toronto Ontario Canada; ^7^ Institute of Health Policy, Management and Evaluation University of Toronto Toronto Ontario Canada; ^8^ Division of General Internal Medicine University Health Network Toronto Ontario Canada

**Keywords:** medical imaging, psychiatric comorbidity, rate of testing, testing delays

## Abstract

**Objective:**

To determine whether presence of a psychiatric comorbidity impacts use of inpatient imaging tests and subsequent wait times.

**Methods:**

This was a retrospective cohort study of all patients admitted to General Internal Medicine (GIM) at five academic hospitals in Toronto, Ontario from 2010 to 2019. Exposure was presence of a coded psychiatric comorbidity on admission. Primary outcome was time to test, as calculated from the time of test ordering to time of test completion, for computed tomography (CT), magnetic resonance imaging (MRI), ultrasound, or peripherally inserted central catheter (PICC) insertion. Multilevel mixed‐effects models were used to identify predictors of time to test, and marginal effects were used to calculate differences in absolute units (h). Secondary outcome was the rate of each type of test included. Subgroup analyses were performed according to type of psychiatric comorbidity: psychotic, mood/anxiety, or substance use disorder.

**Results:**

There were 196,819 GIM admissions from 2010to 2019. In 77,562 admissions, ≥1 advanced imaging test was performed. After adjusting for all covariates, presence of any psychiatric comorbidity was associated with increased time to test for MRI (adjusted difference: 5.3 h, 95% confidence interval [CI]: 3.9–6.8), PICC (adjusted difference: 3.7 h, 95% CI: 1.6–5.8), and ultrasound (adjusted difference: 3.0 h, 95% CI: 2.3–3.8), but not for CT (adjusted difference: 0.1 h, 95% CI: −0.3 to 0.5). Presence of any psychiatric comorbidity was associated with lower rate of ordering for all test types (adjusted difference: −17.2 tests per 100 days hospitalization, interquartile range: −18.0 to −16.3).

**Conclusions:**

There was a lower rate of ordering of advanced imaging among patients with psychiatric comorbidity. Once ordered, time to test completion was longer for MRI, ultrasound, and PICC. Further exploration, such as quantifying rates of cancelled tests and qualitative studies evaluating hospital, provider, and patient barriers to timely advanced imaging, will be helpful in elucidating causes for these disparities.

## Graphical abstract

This was a retrospective cohort study of all patients admitted to General Internal Medicine at five academic hospitals in Toronto, Ontario from 2010 to 2019. There was a lower rate of ordering of advanced imaging among patients with psychiatric comorbidity; furthermore, once ordered, time to test completion was longer for MRI, ultrasound, and PICC. Further exploration, such as quantifying rates of cancelled tests and qualitative studies evaluating hospital, provider, and patient barriers to timely advanced imaging, will be helpful in elucidating causes for these disparities.

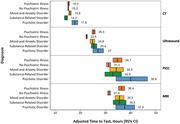

## 1 BACKGROUND

There is a bidirectional association between mental and physical health disorders, such that individuals with mental health disorders are at greater risk of common chronic medical conditions, such as hypertension, diabetes, and chronic obstructive pulmonary disease (Lin et al., 2011; Scott et al., 2016; Sporinova et al., 2019; Surveillance & Epidemiology Division, Public Health Agency of Canada et al., 2015). A study of individuals from 17 countries demonstrated an independent association between mental and physical health conditions; for example, depression was associated with an increased odds of subsequent onset of hypertension, arthritis, and lung disease (Scott et al., 2016). Individuals with chronic medical conditions also have an increased risk of psychiatric conditions like depression and anxiety (Sporinova et al., 2019; Surveillance & Epidemiology Division, Public Health Agency of Canada et al., 2015). A study of nearly 1 million Canadians with common chronic medical conditions showed that 15.8% had a concomitant mental health disorder (Sporinova et al., 2019). Psychiatric illnesses are independently associated with increased risk of cardio‐ and cerebrovascular disease (Lambert et al., 2022; Vance et al., 2019). Patients with psychiatric comorbidities, compared with those without, have increased medical costs, longer length of stay, and higher rates of readmission, nosocomial infection, and postoperative complications (Beeler et al., 2020; Bressi et al., 2006; Chwastiak et al., 2014; Daratha et al., 2012; Daumit et al., 2006; Druss et al., 2001; Jansen et al., 2018; Sporinova et al., 2019).

In addition, patients with psychiatric illness may be less likely to receive standard treatments for common medical conditions (Daumit et al., 2006; Druss et al., 2001). Patients with psychiatric illness were less likely to receive reperfusion therapy and guideline‐based medical therapy (i.e., aspirin, beta‐blocker, and angiotensin‐converting enzyme inhibitor) after a myocardial infarction (Druss et al., 2001), had higher adjusted mortality rates, and were less likely to follow up with a cardiologist (Kurdyak et al., 2012). Similarly, patients with stroke and psychiatric comorbidity have lower rates of thrombolysis, carotid artery revascularization, rehabilitation attendance, and treatment with antiplatelet and lipid‐lowering medications (Bongiorno et al., 2018, 2019; Kapral et al., 2021).

Advanced imaging tests and radiological procedures, including computed tomography (CT), magnetic resonance imaging (MRI), ultrasound, and peripherally inserted central catheter (PICC) insertion, are commonly used to aid in both diagnosis and management of medical inpatients (Smith‐Bindman et al., 2019; Verma et al., 2017). Given the finite resources in hospital, wait times for these tests can be prolonged (Cournane et al., 2016). While there is ample evidence that the care provided in hospital to patients with psychiatric illness is different than for patients without psychiatric illness, this is the first study, to our knowledge, to evaluate whether the use of inpatient imaging and associated waiting time is impacted by the presence of a psychiatric comorbidity. We have previously shown that delays in advanced imaging are associated with longer length of stay in hospital (Bartsch et al., 2023); therefore, disparities in wait times for advanced imaging between patients with and without psychiatric comorbidity could serve as one explanation for longer length of stay observed among patients with psychiatric comorbidity.

The objective of our study was to determine whether the use of advanced imaging, as well as the subsequent waiting time, differs between patients with and without a coded psychiatric diagnosis. Previous studies have shown that patients with psychiatric comorbidity experience differences in their hospitalization, ranging from likelihood of receiving guideline‐based treatments to length of stay (Beeler et al., 2020; Bressi et al., 2006; Daumit et al., 2006; Druss et al., 2001; Sporinova et al., 2019); however, it has yet to be established whether medical imaging is yet another dimension of care that is impacted by the presence of a psychiatric comorbidity. Identifying differences may suggest an additional inequity in patient care and resource allocation, and underline the need to explore causes and potential solutions for this disparity.

## 2 METHODS

### 2.1 Setting, study design, and data sources

We completed a retrospective cohort study of patients admitted to General Internal Medicine (GIM) in Toronto, Ontario, using the General Internal Medicine Inpatient Initiative (GEMINI) database, which includes all admissions to GIM at five academic hospitals affiliated with the University of Toronto (Verma et al., 2017).

GEMINI contains patient‐level clinical data from each hospital, including information that is coded for submission to the Canadian Institute for Health Information Discharge Abstract Database (CIHI‐DAD). The combined data set includes demographics, diagnoses, interventions, discharge destination, resource utilization, laboratory and radiologic testing, pharmacy, and room transfer data (Canadian Institute for Health Information, 2015). The “most responsible physician” (MRP) for each admission is determined by CIHI‐DAD coding standards as the attending physician who is “responsible for the care and treatment of the patient for the majority of the visit to the health care facility” (Canadian Institute for Health Information, 2015). We obtained information on physician characteristics from the College of Physicians and Surgeons of Ontario physician information database, which is publicly available (College of Physicians & Surgeons of Ontario, 2020).

### 2.2 Participants

The cohort included all patients admitted to and discharged from the GIM service between April 1, 2010 and December 31, 2019 at the five academic health centers included within GEMINI.

### 2.3 Exposure

The primary exposure was having a coded psychiatric comorbidity on admission (Canadian Institute for Health Information, 2015). We utilized a definition generated by Ontario Health (formerly Health Quality Ontario) to measure mental health and addictions‐related health service utilization (Brien et al., 2015; Health Quality Ontario, n.d.). The International Statistical Classification of Diseases and Related Health Problems (ICD) 10 codes for psychiatric comorbidity, captured in CIHI‐DAD, are as follows: all F04‐F99, substance‐related disorders (F55, F10–F19), psychotic disorders, not limited to schizophrenia (F20–F29), mood disorders (F30–F34, F38, F39, F53.0), and anxiety disorders (F40–F43, F48.8, F48.9) (Canadian Institute for Health Information, 2015; Doktorchik et al., 2019; Kurdyak et al., 2015).

### 2.4 Outcome measures

The primary outcome was time to test, in hours, as calculated from the time of ordering to the time of test completion. The secondary outcome was the rate of each type of included test, per 100 patient‐days. Our data set did not include any tests that were ordered but not completed (cancelled).

### 2.5 Other variables

We included test‐, admission‐, and physician‐level variables expected to affect the decision to order advanced imaging, and the subsequent wait time to test in hospital. Test‐level variables included type of test/procedure (CT, MRI, ultrasound, or PICC insertion), time ordered relative to time of admission, setting and timing of test ordering (emergency department, ICU, or ward; whether ordered on weekdays or during evenings and weekends), and GIM total patient census on the day of test ordering. Admission‐level variables included the patient age, sex, neighborhood income quintile, most responsible diagnosis (based on the Clinical Classifications Software Refined tool that aggregates ICD‐10 codes into 285 distinct clinical diagnoses [Agency for Healthcare Research and Quality, 2017]), Charlson comorbidity index, laboratory‐based acute physiology score (LAPS), and fiscal year of admission (Escobar et al., 2008; Quan et al., 2011; Statistics Canada Catalogue, 2017). Physician‐level variables included gender, years in practice, whether the MRP was a general internist/hospitalist versus a subspecialist, and their annual GIM patient volume.

### 2.6 Statistical analysis

Demographic data were expressed as means with standard deviations (SD), counts, and frequencies. We compared patients with and without a coded psychiatric comorbidity using standardized mean differences (SMDs) given large sample sizes in GEMINI. An SMD >0.1 is considered meaningful (Austin, 2009). We reported the use of advanced imaging based on the volume of tests ordered, test type, tests per patient, time to test completion, time from admission to test ordering, and the proportion of hospital days spent waiting for a test.

For each of the four test types (CT, MRI, ultrasound, or PICC), we used separate mixed‐effect negative binomial models with random intercepts at the admission, patient, and physician levels (with crossed random intercepts between patients and physicians) to measure the association between psychiatric comorbidity and time to test. Random intercepts accounted for clustering of tests at the admission, patient, and physician levels. Patients who had at least one advanced imaging test performed after admission were included in this analysis (Figure S1). We performed prespecified stratified analyses according to type of psychiatric comorbidity (mood and anxiety disorders, psychotic disorders, and substance‐related disorders).

We reported adjusted time to test as a difference in hours and adjusted rate of tests ordered, or average marginal effects, obtained using the “margins” package in R (CRAN, 2021). Average marginal effects are calculated as the mean of partial derivatives of the regression model with respect to each variable and each observation in the data—in contrast with marginal effects at the mean, average marginal effects have the advantage of keeping all other variables at their naturally observed values (Norton et al., 2019).

Multivariable Poisson models with the log of length of stay as an offset were also used to identify predictors of the rate of tests ordered per 100 days hospitalized, the secondary outcome. The cohort in this analysis included all admissions, regardless of whether a test was ordered. For patients who had any days designated as alternate level of care (ALC) (Health Analytics Branch, Ministry of Health and Long‐Term Care, 2017), which are days spent waiting for a discharge destination, we subtracted the number of ALC days from the total “days hospitalized” denominator for this outcome. As ALC days are, by definition, periods when patients are medically inactive, we removed these days from our calculation to avoid artificially lowering the rate of tests ordered.

All statistical analyses were performed using R version 4.0.2.

### 2.7 Ethics approval

Research Ethics Board approval was obtained from St. Michael's Hospital on behalf of all participating hospitals (Study ID 15–087), with a waiver of patient consent for this retrospective study using routinely collected health data.

## 3 RESULTS

### 3.1 Study cohort

From April 1, 2010 to December 31, 2019, 196,819 patients were admitted to GIM. About half (49.2%) were women and the median age was 71 years old (Table 1). A total of 18.4% had a comorbid psychiatric illness; the most common of these were mood and anxiety disorders (*n* = 17,345, 47.8%). Patients with psychiatric comorbidity tended to be younger (median age: 65 years old, interquartile range [IQR]: 50–80) than patients without psychiatric comorbidity (median age: 72 years old, IQR: 56–83), although the number of comorbidities, based on Charlson score, was similar between groups. Acute length of stay was longer (SMD: 0.14) for patients with psychiatric comorbidity (median: 115.0 h, IQR: 60.0–223.0) than for those without (median: 96.0 h, IQR: 50.0–182.0). Proportion of the admission spent waiting for advanced imaging tests was similar for patients with (mean: 8%, SD: 13%) and without (mean: 10%, SD: 14%) psychiatric comorbidity.

**TABLE 1:** Baseline characteristics of patients admitted to General Internal Medicine at five academic hospitals in Toronto, Ontario from April 1, 2010 to December 31, 2019.

Characteristic	Patients with psychiatric comorbidity(*n* = 36,309)	Patients with no psychiatric comorbidity(*n* = 160,510)	SMD
Sex, male	18,765 (51.7)	81,159 (50.6)	0.02
Age, years			0.26
18–39	5113 (14.1)	16,349 (10.2)	
40–64	12,770 (35.2)	42,765 (26.6)	
65–79	8956 (24.7)	45,692 (28.5)	
80 or older	9470 (26.1)	55,704 (34.7)	
Charlson Comorbidity Index score			0.16
0	14,666 (40.4)	60,666 (37.8)	
1	5453 (15.0)	24,869 (15.5)	
2	7016 (19.3)	30,821 (19.2)	
3	3411 (9.4)	15,522 (9.7)	
≥4	5763 (15.9)	28,632 (17.8)	
LAPS, median (IQR)	12.0 (5.0, 24.0)	14.0 (5.0, 26.0)	0.06
Top 10 most responsible diagnoses			
Chronic obstructive pulmonary disease	1878 (5.2)	7037 (4.4)	0.03
Pneumonia	1860 (5.1)	8520 (5.4)	0.01
Urinary tract infection	1633 (4.5)	6319 (4.0)	0.03
Congestive heart failure	1249 (3.4)	8548 (5.4)	0.10
Neurocognitive disorders	1163 (3.2)	4039 (2.5)	0.04
Gastrointestinal bleed	970 (2.7)	5015 (3.2)	0.03
Sepsis	713 (2.0)	3358 (2.1)	0.01
Acute and unspecified renal failure	621 (1.7)	2891 (1.8)	0.01
Cerebral infarction	541 (1.5)	3479 (2.2)	0.05
Secondary malignancies	265 (0.7)	3028 (1.9)	0.10
Time spent waiting for test, days, median (IQR)	0.9 (0.2, 2.2)	0.8 (0.2, 1.9)	0.12
Proportion of days spent waiting for test, days, median (IQR)	0.1 (0.0, 0.2)	0.1 (0.0, 0.2)	0.08
Length of stay, days, median (IQR)	5.1 (2.5, 11.0)	4.3 (2.1, 8.3)	0.15
At least one advanced imaging test performed during admission	15,168 (41.8)	62,394 (38.9)	0.06
At least one test ordered from GIM	7429 (20.5)	29,945 (18.7)	0.05
At least one test ordered from ICU	1063 (2.9)	3612 (2.3)	0.04
At least one test ordered from ED	6233 (17.2)	24,934 (15.5)	0.04
At least one test ordered while bedspaced	3761 (10.4)	15,760 (9.8)	0.02
At least one test ordered on weekend	5006 (13.8)	19,704 (12.3)	0.05
At least one test ordered at night	7444 (20.5)	30,075 (18.7)	0.04
Number of tests, mean (SD)	0.8 (1.5)	0.7 (1.3)	0.07
Number of tests during admission			0.08
None	21,141 (58.2)	98,116 (61.1)	
One	8863 (24.4)	38,056 (23.7)	
Two	3385 (9.3)	14,034 (8.7)	
Three	1410 (3.9)	5457 (3.5)	
Four or more	1510 (4.2)	4847 (3.0)	
Tests during admission, mean (SD)			
CT	0.43 (1.04)	0.38 (0.90)	0.05
MRI	0.09 (0.35)	0.07 (0.31)	0.05
Ultrasound	0.24 (0.56)	0.21 (0.52)	0.05
PICC	0.05 (0.27)	0.04 (0.24)	0.03
Fiscal year of admission			0.12
2010	2747 (7.6)	15,226 (9.5)	
2011	2854 (7.9)	16,165 (10.1)	
2012	3395 (9.4)	15,866 (9.9)	
2013	3757 (10.3)	16,095 (10.0)	
2014	3932 (10.8)	16,951 (10.6)	
2015	4040 (11.1)	16,902 (10.5)	
2016	4161 (11.5)	16,942 (10.6)	
2017	4309 (11.9)	17,409 (10.8)	
2018	4407 (12.1)	17,163 (10.7)	
2019	2707 (7.5)	11,791 (7.3)	
Neighborhood income quintile^a^			0.14
Q1	8724 (24.0)	33,405 (21.4)	
Q2	6036 (16.6)	27,039 (16.8)	
Q3	5388 (14.8)	25,502 (15.9)	
Q4	5107 14.1)	24,050 (15.0)	
Q5	5555 (15.3)	30,190 (18.8)	
Information not available	5499 (15.1)	19,324 (12.0)	
Neighborhood deprivation quintile^b^			0.10
Q1	8217 (22.6)	37,813 (23.6)	
Q2	4899 (13.5)	23,881 (14.9)	
Q3	4542 (12.5)	21,943 (13.7)	
Q4	5624 (15.5)	26,034 (16.2)	
Q5	7446 (20.5)	31,027 (19.3)	
Information not available	5581 (15.4)	19,812 (12.3)	

Abbreviations: CT, computed tomography; ED, emergency department; GIM, general internal medicine ward; ICU, intensive care unit; IQR, interquartile range; LAPS, laboratory‐based acute physiology score; MRI, magnetic resonance imaging; PICC, peripherally inserted central catheter; SMD, standardized mean difference.

^a^ Q1 is lowest income and Q5 is highest income.

^b^ Q1 is highest level of deprivation and Q5 is lowest level of deprivation.

### 3.2 Testing volumes

A total of 41.8% of patients with psychiatric comorbidity had at least one advanced imaging test performed during admission, which was similar for patients without psychiatric comorbidity (38.9%, SMD: 0.06) (Table 1). The mean number of tests performed during admission was similar (SMD: 0.07) for patients with psychiatric comorbidity (mean: 0.81, SD: 1.53) than for those without (mean: 0.70, SD: 1.33) (Table 1).

Across both groups, the most common test ordered was CT (51.5% of all tests for patients with psychiatric comorbidity and 52.1% for patients without), followed by ultrasound (30.5% of all tests for patients with psychiatric comorbidity and 30.9% for patients without) (Table 2). More tests were ordered within the first day of admission for patients without psychiatric comorbidity than for patients with psychiatric comorbidity (46.7% of all tests vs. 41.5%, respectively, SMD: 0.15). In contrast, patients with psychiatric comorbidity had more tests ordered later into the admission, after at least 4 days, than patients with no psychiatric comorbidity (36.5% of all tests vs. 29.7%, respectively, SMD: 0.15).

**TABLE 2:** Tests ordered for patients with psychiatric comorbidity, including by subgroup of psychiatric illness, and without psychiatric comorbidity.

Characteristic	Patients with psychiatric comorbidity(*n* = 28,438)	Patients with no psychiatric comorbidity (*n* = 109,555)	SMD
Test type			0.03
CT	14,651 (51.5)	57,063 (52.1)	
MRI	3163 (11.1)	11,463 (10.5)	
PICC	1940 (6.8)	7190 (6.6)	
Ultrasound	8684 (30.5)	33,839 (30.9)	
CT by anatomical region			
Brain	5867 (20.6)	17,840 (16.3)	0.11
Thorax	4347 (15.3)	20,446 (18.7)	0.09
Abdomen/pelvis	4663 (16.4)	19,995 (18.3)	0.05
MRI by anatomical region			
Brain	2364 (8.3)	8571 (7.8)	0.02
Spine	842 (3.0)	3163 (2.9)	0.00
Ultrasound by anatomical region			
Venous leg with Doppler	2288 (8.0)	10,512 (9.6)	0.06
Abdomen	5942 (20.9)	21,949 (20.0)	0.02
Extremity	469 (1.6)	1498 (1.4)	0.02
Time from admission to ordering test			0.15
Less than 1 day	11,789 (41.5)	51,133 (46.7)	
1–2 days	2742 (9.6)	11,920 (10.9)	
2–4 days	3520 (12.4)	13,990 (12.8)	
4 or more days	10,387 (36.5)	32,512 (29.7)	
Median time from test ordering to completion, h (IQR)	12.0 (2.4, 28.8)	12.0 (2.4, 26.4)	0.06
Median time spent waiting for tests, h (IQR)	13.1 (3.0, 27.7)	12.4 (3.1, 26.0)	0.06
Proportion of total days spent waiting for tests, mean (SD)	0.1 (0.1)	0.1 (0.1)	0.12
Tests ordered in emergency department	7372 (25.9)	29,524 (26.9)	0.02
Tests ordered in intensive care unit	2216 (7.8)	7340 (6.7)	0.04
Tests ordered during the weekend	9686 (34.1)	38,518 (35.2)	0.02
Tests ordered overnight	6460 (22.7)	25,114 (22.9)	0.01
Tests ordered while patient was bedspaced	6445 (22.7)	25,537 (23.3)	0.02
GIM census, median (IQR)	94.0 (84.0, 108.0)	95.0 (84.0, 111.0)	0.09
Capacity ratio, median (IQR)	1.0 (0.9, 1.1)	1.0 (0.9, 1.1)	0.04
Income quintile^a^			0.13
Q1	6912 (24.3)	23,535 (21.5)	
Q2	4653 (16.4)	18,197 (16.6)	
Q3	4231 (14.9)	17,400 (15.9)	
Q4	3845 (13.5)	16,904 (15.4)	
Q5	4288 (15.1)	19,514 (17.8)	
Information not available	4509 (15.9)	14,005 (12.8)	
Deprivation quintile^b^			0.10
Q1	6154 (21.6)	24,025 (21.9)	
Q2	3810 (13.4)	15,851 (14.5)	
Q3	3576 (12.6)	15,575 (14.2)	
Q4	4398 (15.5)	17,811 (16.3)	
Q5	5939 (20.9)	21,939 (20.0)	
Information not available	4561 (16.0)	14,354 (13.1)	

*Note*: Values are numbers (percentages) unless stated otherwise.

Abbreviations: CT, computed tomography; IQR, interquartile range; MRI, magnetic resonance imaging; PICC, peripherally inserted central catheter; SMD, standardized mean difference.

^a^ Q1 is lowest income and Q5 is highest income.

^b^ Q1 is highest level of deprivation and Q5 is lowest level of deprivation.

CT brain was performed more commonly for patients with psychiatric comorbidity than for patients without psychiatric comorbidity (20.6% of all tests vs. 16.3%, SMD: 0.11) (Table 2). Compared to patients with no psychiatric comorbidity, patients with psychotic illnesses were more likely to undergo CT brain (20.0% of all tests vs. 16.3%, SMD 0.10) (Table S1a). Compared to patients without psychiatric comorbidity, patients with substance‐related disorders underwent more abdominal ultrasounds (28.2% of all tests vs. 20.0%, SMD: 0.19) and fewer thoracic CTs (13.9% of all tests vs. 18.7%, SMD: 0.13) (Table S1b). For all test types, there were no significant differences between patients with mood and anxiety disorders and patients without psychiatric comorbidity (SMD: 0.05) (Table S1c).

### 3.3 Time to test

**FIGURE 1:**
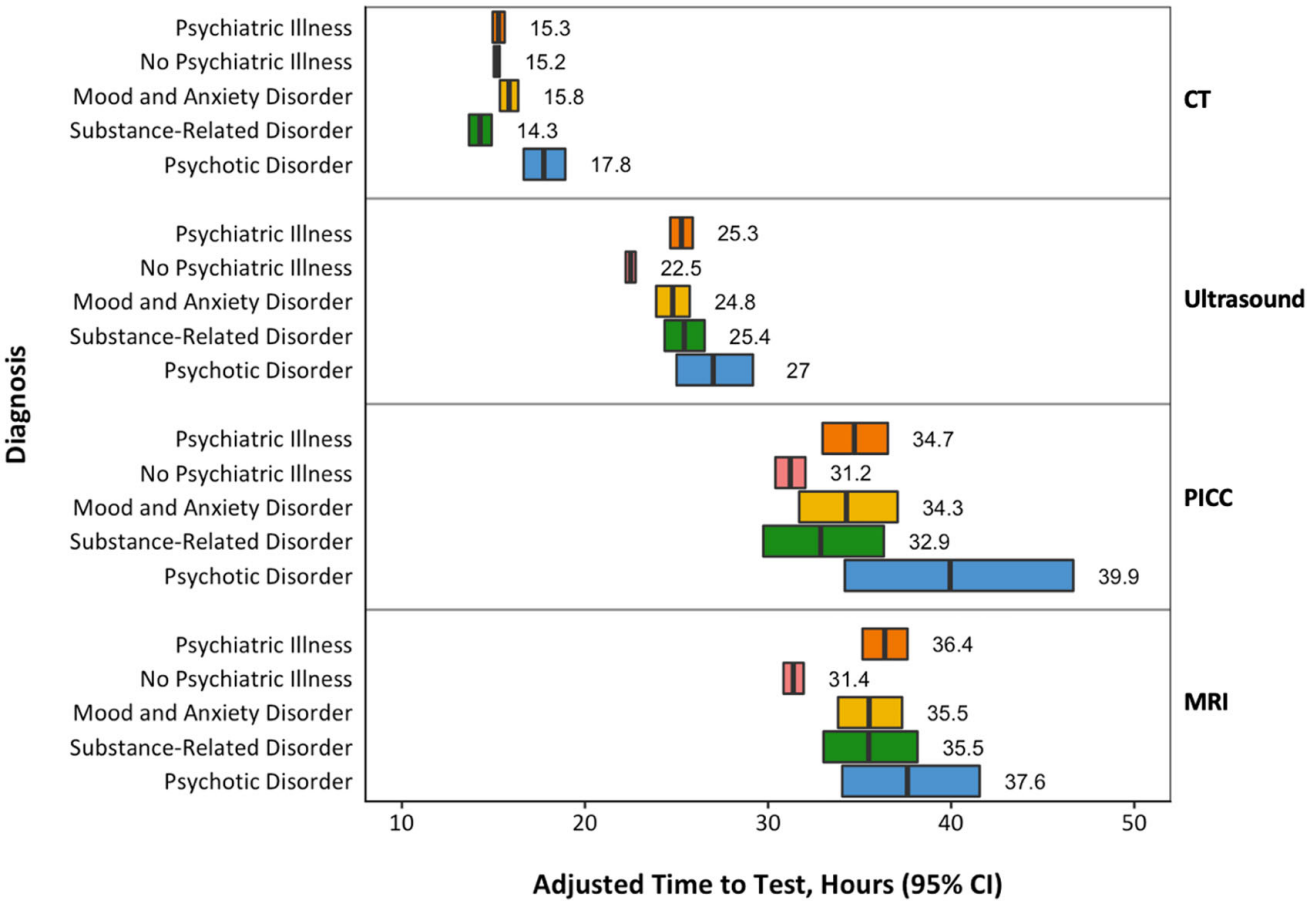
Adjusted time to test (h, 95% confidence interval) comparing patients with any psychiatric comorbidity and with specific psychiatric comorbidity (substance use disorder, mood and anxiety disorder, and psychotic disorder) to patients with no psychiatric comorbidity. CT, computed tomography; PICC, peripherally inserted central catheter; MRI, magnetic resonance imaging.

Unadjusted wait times for all radiological procedures were similar between patients with (median: 12.0 h, IQR: 2.4–28.8) and without (median: 12.0 h, IQR: 2.4–26.4) psychiatric comorbidity. After adjusting for all other variables, presence of any psychiatric comorbidity was associated with increased time to test for MRI (adjusted difference: 5.3 h, 95% confidence interval [CI]: 3.9–6.8), PICC (adjusted difference: 3.7 h, 95% CI: 1.6–5.8), and ultrasound (adjusted difference: 3.0 h, 95% CI: 2.3–3.8), but not for CT (adjusted difference: 0.1 h, 95% CI: −0.28 to 0.5) (Figure 1).

### 3.4 Psychiatric subgroup analyses

Compared to patients with no psychiatric comorbidity, patients with psychotic disorders experienced significantly increased time to test for CT (adjusted difference: 2.7 h, 95% CI: 1.5–4.0). In addition, the magnitudes of delay were accentuated in the psychotic illness group for PICC (adjusted difference: 9.1 h, 95% CI: 2.5–15.7), MRI (adjusted difference: 7.1 h, 95% CI: 3.1–11.2), and ultrasound (adjusted difference: 4.9 h, 95% CI: 2.6–7.1) (Figure 1). Adjusted time to test for patients with mood and anxiety disorders was increased for MRI (adjusted difference: 4.7 h, 95% CI: 2.7–6.6) and ultrasound (adjusted difference: 2.5 h, 95% CI: 1.4–3.5). In contrast, presence of a substance‐related disorder was associated with reduced time to test for CT (adjusted difference: −1.0 h, 95% CI: −1.7 to −0.3), while increased time to test was observed for both MRI (adjusted difference: 5.0 h, 95% CI: 2.2–7.8) and ultrasound (adjusted difference: 3.3 h, 95% CI: 2.0–4.5).

### 3.5 Rate of testing

The unadjusted rate of testing was not significantly different between patients with and without a psychiatric comorbidity (7.0 per 100 patient‐days hospitalized vs. 8.9 per 100 patient‐days hospitalized, respectively, *p* < .001) (Table 2). After adjusting for all covariates, the presence of any psychiatric comorbidity was associated with a reduced rate of testing for all test types (adjusted difference: −17.2 tests per 100 days hospitalized, IQR: −18.0 to −16.3), as well as for each individual test type: CT (adjusted difference: −9.1 tests per 100 days hospitalized, IQR: −9.7 to −8.5), ultrasound (adjusted difference: −5.4 tests per 100 days hospitalized, IQR: −5.8 to −4.9), MRI (adjusted difference: −2.0 tests per 100 days hospitalized, IQR: −2.4 to −1.7), and PICC (adjusted difference: −1.1 tests per 100 days hospitalized, IQR: −1.3 to −0.9).

## 4 DISCUSSION

In this population‐based cohort study of GIM hospitalizations, we found that presence of any psychiatric comorbidity was associated with increased wait times for advanced imaging, and that this effect was most pronounced in patients with psychotic illness. Furthermore, the rate of test ordering for all test types was lower for patients with psychiatric comorbidity.

Most patients with psychiatric illness have comorbid medical conditions, and the presence of psychiatric illness is associated with increased healthcare utilization and costs (longer length of stay, higher readmission rates) (Bressi et al., 2006; Chwastiak et al., 2014; Daratha et al., 2012; Jansen et al., 2018; Sporinova et al., 2019; Sprah et al., 2017). Furthermore, there is ample evidence that patients with psychiatric comorbidity experience more adverse outcomes and receive differential treatment in hospital, as compared to patients without psychiatric illness (Bongiorno et al., 2019; Daumit et al., 2006; Druss et al., 2001; Kurdyak et al., 2012). As examples, such patients are less likely to undergo carotid revascularization following stroke and less likely to receive reperfusion therapy after myocardial infarction (Bongiorno et al., 2018, 2019; Kurdyak et al., 2012).

Our study demonstrates that radiological procedures are another facet of care that is impacted by the presence of psychiatric illness. We found that having a coded psychiatric comorbidity was associated with significantly longer wait times for MRI, PICC, and ultrasound. This effect was amplified in patients with psychotic illness, who experienced delays for all test types, and with greater degrees of delay. This may reflect increased severity of mental illness in patients with psychosis, and subsequent challenges in obtaining consent and completing advanced imaging tests. In contrast to our general finding of increased wait times for patients with psychiatric comorbidity, the presence of a substance use disorder was associated with decreased time to test for CT. This may be related to presentation with decreased level of consciousness or altered mental status in the setting of substance use, for which urgent neurological imaging is often performed.

We observed longer wait times for patients with psychiatric comorbidity. Furthermore, there was a lower rate of testing among patients with psychiatric comorbidity, which may reflect more cancelled tests in this patient group, as opposed to systematic discrepancies in ordering practice. Unfortunately, our data sources only included completed tests. It is well established that inpatients with psychiatric comorbidity are more likely to be prematurely discharged ( Brook et al., 2006; Simon et al., 2020). One qualitative study of patients with substance use disorders, for example, elucidated specific reasons for self‐discharge, which included undertreated withdrawal, ongoing substance use cravings, and feeling discriminated against and stigmatized by the healthcare team (Simon et al., 2020). In light of these findings, it is foreseeable that patients with psychiatric comorbidity may be less likely to proceed with a test—for example, due to anxiety, paranoia, or claustrophobia. Interestingly, provider variables have also been shown to impact likelihood of premature discharge (Doktorchik et al., 2019). Extrapolating these to completion of inpatient imaging tests, limitations in orienting patients to the plan for hospitalization and establishing a supportive patient–provider relationship may decrease the likelihood of patients having tests completed expediently (Brook et al., 2006). To help address disparities between patients with and without psychiatric comorbidity, rates of cancelled tests should be quantified and qualitative studies exploring mechanisms for cancelled, delayed, and re‐ordered tests should be conducted.

This study employed a large, population‐based sample, which enabled characterization of the impact of any psychiatric comorbidity, as well as specific subtypes of psychiatric illness, on the use of advanced imaging and subsequent wait times at five academic hospitals in Toronto, Ontario. As such, our findings are generalizable to other urban centers. Some limitations of our study warrant emphasis. First, the indication for imaging tests ordered was unavailable. Given tests with urgent indications are prioritized, we attempted to mitigate this unknown by accounting for illness severity (using the LAPS) and most common admission diagnoses. Second, our data sources only captured completed tests and did not include tests that were ordered and subsequently canceled. This may have resulted in falsely shortened wait times—for example, when a test is cancelled and re‐ordered by the medical imaging department at the time of test. Third, we used ICD‐10‐CA codes to identify patients with psychiatric illness. Previous studies reported that using ICD codes, in comparison to the reference standard of chart review, has excellent specificity but only low or moderate sensitivity in detecting depression ( Doktorchik et al., 2019; Fiest et al., 2014). Another study calculated positive and negative predictive values of 77% and 76%, respectively, for various psychiatric illnesses detected by ICD‐9 codes, with the reference being patient self‐reported mental health condition (Frayne et al., 2010). Missed identification of patients with psychiatric comorbidity could therefore be diluting effect sizes in our study. We did not have information about the duration or severity of psychiatric illness, which presumably could impact patient ability and/or willingness to communicate symptoms and participate in tests. Lastly, we were unable to report important information that was not readily available in electronic clinical and administrative data, such as smoking status. Presence of smoking may be an indication for imaging (i.e., to evaluate for malignancy) and is also a reason patients may leave hospital with tests pending or may not be in their room when called to imaging tests.

In summary, our study shows that presence of a psychiatric comorbidity is associated with increased wait times for common advanced imaging tests. For all test types studied, we identified a lower testing rate for patients with coded psychiatric comorbidities compared to those without. Further exploration, such as quantifying rates of cancelled tests and qualitative studies evaluating hospital, provider, and patient barriers to timely advanced imaging, will be helpful in elucidating causes for these disparities.

## AUTHOR CONTRIBUTIONS

*Conception and design of the work*: Lauren Lapointe‐Shaw, Emily Bartsch, Kathleen Sheehan. *Acquisition, analysis*: Lauren Lapointe‐Shaw, Emily Bartsch, Saeha Shin, Fahad Razak, Amol Verma. *Interpretation of data for the work*: Lauren Lapointe‐Shaw, Emily Bartsch, Saeha Shin, Kathleen Sheehan. *Drafting the work*: Lauren Lapointe‐Shaw, Emily Bartsch. *Revising it critically for important intellectual content*: All authors. *Final approval of the version to be published*: All authors.

## CONFLICT OF INTEREST STATEMENT

A.V. and F.R. are part‐time employees of Ontario Health, outside of the submitted work. The other authors declare no conflicts of interest.

### PEER REVIEW

The peer review history for this article is available at https://publons.com/publon/10.1002/brb3.3425.

## Supporting information

Supplemental table 1a. Tests ordered for patients with psychotic disorders and without psychiatric comorbidity. Values are numbers (percentages) unless stated otherwise.
Supplemental table 1b. Tests ordered for patients with substance use disorders and without psychiatric comorbidity. Values are numbers (percentages) unless stated otherwise.
Supplemental table 1c. Tests ordered for patients with mood and anxiety disorders and without psychiatric comorbidity. Values are numbers (percentages) unless stated otherwise.
Supplemental figure 1. Creation of the cohort evaluated in the multivariable model.

## Data Availability

We are unable to provide unlimited open access to GEMINI data because of data sharing agreements and research ethics board protocols with participating hospitals. However, researchers can request access to GEMINI data through an established process approved by our institutional research ethics boards. Please see this link for full details: https://www.geminimedicine.ca/access‐data.

## References

[brb33425-bib-0001] Agency for Healthcare Research and Quality . (2017). Healthcare Cost and Utilization Project: Beta Clinical Classifications Software (CCS) for ICD‐10‐CM/PCS . https://www.hcup‐us.ahrq.gov/toolssoftware/ccs10/ccs10.jsp

[brb33425-bib-0002] Austin, P. C. (2009). Using the standardized difference to compare the prevalence of a binary variable between two groups in observational research. Communications in Statistics—Simulation and Computation, 38(6), 1228–1234. 10.1080/03610910902859574

[brb33425-bib-0003] Bartsch, E. , Shin, S. , Roberts, S. , Macmillan, T. E. , Fralick, M. , Liu, J. J. , Tang, T. , Kwan, J. L. , Weinerman, A. , Verma, A. A. , Razak, F. , & Lapointe‐Shaw, L. (2023). Imaging delays among medical inpatients in Toronto, Ontario: A cohort study. PLoS ONE, 18(2), Article e0281327. 10.1371/journal.pone.0281327 36735736 PMC9897551

[brb33425-bib-0004] Beeler, P. E. , Cheetham, M. , Held, U. , & Battegay, E. (2020). Depression is independently associated with increased length of stay and readmissions in multimorbid inpatients. European Journal of Internal Medicine, 73, 59–66. 10.1016/j.ejim.2019.11.012 31791574

[brb33425-bib-0005] Bongiorno, D. M. , Daumit, G. L. , Gottesman, R. F. , & Faigle, R. (2018). Comorbid psychiatric disease is associated with lower rates of thrombolysis in ischemic stroke. Stroke; A Journal of Cerebral Circulation, 49(3), 738–740. 10.1161/STROKEAHA.117.020295 PMC5829001 29374106

[brb33425-bib-0006] Bongiorno, D. M. , Daumit, G. L. , Gottesman, R. F. , & Faigle, R. (2019). Patients with stroke and psychiatric comorbidities have lower carotid revascularization rates. Neurology, 92(22), e2514–e2521. 10.1212/WNL.0000000000007565 31053663 PMC6556087

[brb33425-bib-0007] Bressi, S. K. , Marcus, S. C. , & Solomon, P. L. (2006). The impact of psychiatric comorbidity on general hospital length of stay. The Psychiatric Quarterly, 77(3), 203–209. 10.1007/s11126-006-9007-x 16958003

[brb33425-bib-0008] Brien, S. , Grenier, L. , Kapral, M. E. , Kurdyak, P. , & Vigod, S. (2015). Taking Stock: A report on the quality of mental health and addictions services in Ontario. Health Quality Ontario and Institute for Clinical Evaluative Sciences.

[brb33425-bib-0009] Brook, M. , Hilty, D. M. , Liu, W. , Hu, R. , & Frye, M. A. (2006). Discharge against medical advice from inpatient psychiatric treatment: A literature review. Psychiatric Services, 57(8), 1192–1198. 10.1176/ps.2006.57.8.1192 16870972

[brb33425-bib-0010] Canadian Institute for Health Information . (2015). DAD Abstracting Manual. Author.

[brb33425-bib-0011] Chwastiak, L. A. , Davydow, D. S. , Mckibbin, C. L. , Schur, E. , Burley, M. , Mcdonell, M. G. , Roll, J. , & Daratha, K. B. (2014). The effect of serious mental illness on the risk of rehospitalization among patients with diabetes. Psychosomatics, 55(2), 134–143. 10.1016/j.psym.2013.08.012 24367898 PMC3997382

[brb33425-bib-0012] College of Physicians and Surgeons of Ontario . (2020). Public information and services: Find a doctor . www.cpso.on.ca/Public‐Information‐Services/Find‐a‐Doctor

[brb33425-bib-0013] Comprehensive R Archive Network (CRAN) . (2021). An introduction to ‘margins’ . https://cran.r‐project.org/web/packages/margins/vignettes/Introduction.html#References

[brb33425-bib-0014] Cournane, S. , Conway, R. , Creagh, D. , Byrne, D. G. , Sheehy, N. , & Silke, B. (2016). Radiology imaging delays as independent predictors of length of hospital stay for emergency medical admissions. Clinical Radiology, 71(9), 912–918. 10.1016/j.crad.2016.03.023 27210242

[brb33425-bib-0015] Daratha, K. B. , Barbosa‐Leiker, C. , H Burley, M. , Short, R. , Layton, M. E. , Mcpherson, S. , Dyck, D. G. , Mcfarland, B. H. , & Tuttle, K. R. (2012). Co‐occurring mood disorders among hospitalized patients and risk for subsequent medical hospitalization. General Hospital Psychiatry, 34(5), 500–505. 10.1016/j.genhosppsych.2012.05.001 22703606

[brb33425-bib-0016] Daumit, G. L. , Pronovost, P. J. , Anthony, C. B. , Guallar, E. , Steinwachs, D. M. , & Ford, D. E. (2006). Adverse events during medical and surgical hospitalizations for persons with schizophrenia. Archives of General Psychiatry, 63(3), 267–272. 10.1001/archpsyc.63.3.267 16520431

[brb33425-bib-0017] Doktorchik, C. , Patten, S. , Eastwood, C. , Peng, M. , Chen, G. , Beck, C. A. , Jetté, N. , Williamson, T. , & Quan, H. (2019). Validation of a case definition for depression in administrative data against primary chart data as a reference standard. BMC Psychiatry, 19(1), Article 9. 10.1186/s12888-018-1990-6 30616546 PMC6323719

[brb33425-bib-0018] Druss, B. G. , Bradford, W. D. , Rosenheck, R. A. , Radford, M. J. , & Krumholz, H. M. (2001). Quality of medical care and excess mortality in older patients with mental disorders. Archives of General Psychiatry, 58(6), 565–572. 10.1001/archpsyc.58.6.565 11386985

[brb33425-bib-0019] Escobar, G. J. , Greene, J. D. , Scheirer, P. , Gardner, M. N. , Draper, D. , & Kipnis, P. (2008). Risk‐adjusting hospital inpatient mortality using automated inpatient, outpatient, and laboratory databases. Medical Care, 46(3), 232–239. 10.1097/MLR.0b013e3181589bb6 18388836

[brb33425-bib-0020] Fiest, K. M. , Jette, N. , Quan, H. , St Germaine‐Smith, C. , Metcalfe, A. , Patten, S. B. , & Beck, C. A. (2014). Systematic review and assessment of validated case definitions for depression in administrative data. BMC Psychiatry, 14, Article 289. 10.1186/s12888-014-0289-5 25322690 PMC4201696

[brb33425-bib-0021] Frayne, S. M. , Miller, D. R. , Sharkansky, E. J. , Jackson, V. W. , Wang, F. , Halanych, J. H. , Berlowitz, D. R. , Kader, B. , Rosen, C. S. , & Keane, T. M. (2010). Using administrative data to identify mental illness: What approach is best? American Journal of Medical Quality: The Official Journal of the American College of Medical Quality, 25(1), 42–50. 10.1177/1062860609346347 19855046

[brb33425-bib-0022] Health Analytics Branch, Ministry of Health and Long‐Term Care . (2017). Alternate level of care (ALC) days . https://www.health.gov.on.ca/en/pro/programs/ris/docs/alternate_level_of_care_days_en.pdf

[brb33425-bib-0023] Health Quality Ontario . (n.d.). Welcome to the Health Quality Ontario Indicator Library . http://indicatorlibrary.hqontario.ca/Indicator/Search/EN

[brb33425-bib-0024] Jansen, L. , Van Schijndel, M. , Van Waarde, J. , & Van Busschbach, J. (2018). Health‐economic outcomes in hospital patients with medical‐psychiatric comorbidity: A systematic review and meta‐analysis. PLoS ONE, 13(3), Article e0194029. 10.1371/journal.pone.0194029 29534097 PMC5849295

[brb33425-bib-0025] Kapral, M. K. , Kurdyak, P. , Casaubon, L. K. , Fang, J. , Porter, J. , & Sheehan, K. A. (2021). Stroke care and case fatality in people with and without schizophrenia: A retrospective cohort study. BMJ Open, 11(6), Article e044766. 10.1136/bmjopen-2020-044766 PMC8194334 34112641

[brb33425-bib-0026] Kurdyak, P. , Lin, E. , Green, D. , & Vigod, S. (2015). Validation of a population‐based algorithm to detect chronic psychotic illness. Canadian Journal of Psychiatry, 60(8), 362–368. 10.1177/070674371506000805 26454558 PMC4542516

[brb33425-bib-0027] Kurdyak, P. , Vigod, S. , Calzavara, A. , & Wodchis, W. P. (2012). High mortality and low access to care following incident acute myocardial infarction in individuals with schizophrenia. Schizophrenia Research, 142(1–3), 52–57. 10.1016/j.schres.2012.09.003 23021899

[brb33425-bib-0028] Lambert, A. M. , Parretti, H. M. , Pearce, E. , Price, M. J. , Riley, M. , Ryan, R. , Tyldesley‐Marshall, N. , Avşar, T. S. , Matthewman, G. , Lee, A. , Ahmed, K. , Odland, M. L. , Correll, C. U. , Solmi, M. , & Marshall, T. (2022). Temporal trends in associations between severe mental illness and risk of cardiovascular disease: A systematic review and meta‐analysis. PLoS Medicine, 19(4), Article e1003960. 10.1371/journal.pmed.1003960 35439243 PMC9017899

[brb33425-bib-0029] Lin, W.‐C. , Zhang, J. , Leung, G. Y. , & Clark, R. E. (2011). Chronic physical conditions in older adults with mental illness and/or substance use disorders. Journal of the American Geriatrics Society, 59(10), 1913–1921. 10.1111/j.1532-5415.2011.03588.x 22091505

[brb33425-bib-0030] Norton, E. C. , Dowd, B. E. , & Maciejewski, M. L. (2019). Marginal effects‐quantifying the effect of changes in risk factors in logistic regression models. JAMA: The Journal of the American Medical Association, 321(13), 1304–1305. 10.1001/jama.2019.1954 30848814

[brb33425-bib-0031] Quan, H. , Li, B. , Couris, C. M. , Fushimi, K. , Graham, P. , Hider, P. , Januel, J.‐M. , & Sundararajan, V. (2011). Updating and validating the Charlson comorbidity index and score for risk adjustment in hospital discharge abstracts using data from 6 countries. American Journal of Epidemiology, 173(6), 676–682. 10.1093/aje/kwq433 21330339

[brb33425-bib-0032] Scott, K. M. , Lim, C. , Al‐Hamzawi, A. , Alonso, J. , Bruffaerts, R. , Caldas‐De‐Almeida, J. M. , Florescu, S. , De Girolamo, G. , Hu, C. , De Jonge, P. , Kawakami, N. , Medina‐Mora, M. E. , Moskalewicz, J. , Navarro‐Mateu, F. , O'neill, S. , Piazza, M. , Posada‐Villa, J. , Torres, Y. , & Kessler, R. C. (2016). Association of mental disorders with subsequent chronic physical conditions: World mental health surveys from 17 countries. JAMA Psychiatry, 73(2), 150–158. 10.1001/jamapsychiatry.2015.2688 26719969 PMC5333921

[brb33425-bib-0033] Simon, R. , Snow, R. , & Wakeman, S. (2020). Understanding why patients with substance use disorders leave the hospital against medical advice: A qualitative study. Substance Abuse: Official Publication of the Association for Medical Education and Research in Substance Abuse, 41(4), 519–525. 10.1080/08897077.2019.1671942 31638862

[brb33425-bib-0034] Smith‐Bindman, R. , Kwan, M. L. , Marlow, E. C. , Theis, M. K. , Bolch, W. , Cheng, S. Y. , Bowles, E. J. A. , Duncan, J. R. , Greenlee, R. T. , Kushi, L. H. , Pole, J. D. , Rahm, A. K. , Stout, N. K. , Weinmann, S. , & Miglioretti, D. L. (2019). Trends in use of medical imaging in US health care systems and in Ontario, Canada, 2000–2016. JAMA: The Journal of the American Medical Association, 322(9), 843–856. 10.1001/jama.2019.11456 31479136 PMC6724186

[brb33425-bib-0035] Sporinova, B. , Manns, B. , Tonelli, M. , Hemmelgarn, B. , Macmaster, F. , Mitchell, N. , Au, F. , Ma, Z. , Weaver, R. , & Quinn, A. (2019). Association of mental health disorders with health care utilization and costs among adults with chronic disease. JAMA Network Open, 2(8), Article e199910. 10.1001/jamanetworkopen.2019.9910 31441939 PMC6714022

[brb33425-bib-0036] Šprah, L. , Dernovšek, M. Z. , Wahlbeck, K. , & Haaramo, P. (2017). Psychiatric readmissions and their association with physical comorbidity: A systematic literature review. BMC Psychiatry, 17(1), Article 2. 10.1186/s12888-016-1172-3 28049441 PMC5210297

[brb33425-bib-0037] Statistics Canada Catalogue . (2017). Postal Code^OM^ Conversion File (PCCF) . Author.

[brb33425-bib-0038] Surveillance and Epidemiology Division, Public Health Agency of Canada, CCDSS Mental Illness Working Group, CCDSS Science Committee, & CCDSS Technical Working Group . (2015). Report Summary–Mental Illness in Canada, 2015. Health Promotion and Chronic Disease Prevention in Canada: Research, Policy and Practice, 35(6), 95–96. 10.24095/hpcdp.35.6.02 26302228 PMC4910466

[brb33425-bib-0039] Vance, M. C. , Wiitala, W. L. , Sussman, J. B. , Pfeiffer, P. , & Hayward, R. A. (2019). Increased cardiovascular disease risk in veterans with mental illness. Circulation: Cardiovascular Quality and Outcomes, 12(10), Article e005563. 10.1161/CIRCOUTCOMES.119.005563 31547692

[brb33425-bib-0040] Verma, A. A. , Guo, Y. , Kwan, J. L. , Lapointe‐Shaw, L. , Rawal, S. , Tang, T. , Weinerman, A. , Cram, P. , Dhalla, I. A. , Hwang, S. W. , Laupacis, A. , Mamdani, M. M. , Shadowitz, S. , Upshur, R. , Reid, R. J. , & Razak, F. (2017). Patient characteristics, resource use and outcomes associated with general internal medicine hospital care: The General Medicine Inpatient Initiative (GEMINI) retrospective cohort study. CMAJ Open, 5(4), E842–E849. 10.9778/cmajo.20170097 PMC5741428 29237706

