# Supplementary material for: Advanced imaging use and delays among inpatients with psychiatric comorbidity
Source: Brain Behav. 2024 Feb 15;14(2):e3425. doi: 10.1002/brb3.3425 (PMC10869880; doi:10.1002/brb3.3425)
Supplement: Supplementary file 1 — Supplemental table 1a. Tests ordered for patients with psychotic disorders and without psychiatric comorbidity. Values are numbers (percentages) unless stated otherwise. Supplemental table 1b. Tests ordered for patients with substance use disorders and without psychiatric comorbidity. Values are numbers (percentages) unless stated otherwise. Supplemental table 1c. Tests ordered for patients with mood and anxiety disorders and without psychiatric comorbidity. Values are numbers (percentages) unless stated otherwise. Supplemental figure 1. Creation of the cohort evaluated in the multivariable model. [file BRB3-14-e3425-s001.docx]

**Supplemental table 1a. Tests ordered for patients with psychotic disorders and without psychiatric comorbidity. Values are numbers (percentages) unless stated otherwise.**

| **Characteristic** | **Patients with psychotic disorders**  **n = 2959** | **Patients with no psychiatric comorbidity**  **n = 109,555** | **SMD** |
| --- | --- | --- | --- |
| **Test type**  CT  MRI  PICC  Ultrasound | 1548 (52.3)  357 (12.1)  212 (7.2)  842 (28.5) | 57063 (52.1)  11463 (10.5)  7190 (6.6)  33839 (30.9) | 0.07 |
| **CT by anatomical region** Brain  Thorax  Abdomen/pelvis | 591 (20.0)  502 (17.0)  479 (16.2) | 17840 (16.3)  20446 (18.7)  19995 (18.3) | 0.07  0.03  0.05 |
| **MRI by anatomical region**  Brain  Spine | 273 (9.2)  89 (3.0) | 8571 (7.8)  3163 (2.9) | 0.05  0.01 |
| **Ultrasound by anatomical region** Venous leg with Doppler  Abdomen  Extremity | 257 (8.7)  545 (18.4)  40 (1.4) | 10512 (9.6)  21949 (20.0)  1498 (1.4) | 0.02  0.05  0.01 |
| **Time from admission to ordering test** Less than 1 day  1-2 days  2-4 days  4 or more days | 1283 (43.4)  287 (9.7)  357 (12.1)  1032 (34.9) | 51133 (46.7)  11920 (10.9)  13990 (12.8)  32512 (29.7) | 0.08 |
| **Median time from test ordering to completion, hours (IQR)** | 14.4 (2.4, 31.2) | 12.0 (2.4, 26.4) | 0.12 |
| **Median time spent waiting for tests, hours (IQR)** | 11.8 (3.0, 27.3) | 12.4 (3.1, 26.0) | 0.12 |
| **Proportion of total days spent waiting for tests, mean (SD)** | 0.1 (0.1) | 0.1 (0.1) | 0.05 |
| **Tests ordered in Emergency Department** | 841 (28.4) | 29524 (26.9) | 0.04 |
| **Tests ordered in Intensive Care Unit** | 216 (7.3) | 7340 (6.7) | 0.02 |
| **Tests ordered during the weekend** | 1061 (35.9) | 38518 (35.2) | 0.02 |
| **Tests ordered overnight** | 700 (23.7) | 25114 (22.9) | 0.02 |
| **Tests ordered while patient was bedspaced** | 621 (21.0) | 25537 (23.3) | 0.05 |
| **GIM census, median (IQR)** | 93.0 (83.0, 104.0) | 95.0 (84.0, 111.0) | 0.21 |
| **Capacity ratio, median (IQR)** | 1.0 (0.9, 1.1) | 1.0 (0.9, 1.1) | 0.04 |
| **Income quintile**^a^ |  |  | 0.38 |
| Q1 | 828 (28.0) | 29619 (21.9) |  |
| Q2 | 553 (18.7) | 22297 (16.5) |  |
| Q3 | 459 (15.5) | 21172 (15.7) |  |
| Q4 | 351 (11.9) | 20398 (15.1) |  |
| Q5 | 205 (6.9) | 23597 (17.5) |  |
| Information Not Available | 563 (19.0) | 17951 (13.3) |  |
| **Deprivation quintile**^b^ |  |  | 0.24 |
| Q1 | 416 (14.1) | 29763 (22.0) |  |
| Q2 | 414 (14.0) | 19247 (14.3) |  |
| Q3 | 446 (15.1) | 18705 (13.9) |  |
| Q4 | 472 (16.0) | 21737 (16.1) |  |
| Q5 | 646 (21.8) | 27232 (20.2) |  |
| Information Not Available | 565 (19.1) | 18350 (13.6) |  |

SMD – standardized mean difference; CT – computed tomography; MRI – magnetic resonance imaging; PICC – peripherally-inserted central catheter; IQR – interquartile range. a – Q1 is lowest income and Q5 is highest income. b – Q1 is highest level of deprivation and Q5 is lowest level of deprivation.

**Supplemental table 1b. Tests ordered for patients with substance use disorders and without psychiatric comorbidity. Values are numbers (percentages) unless stated otherwise.**

| **Characteristic** | **Patients with substance use disorders**  **n = 7685** | **Patients with no psychiatric comorbidity**  **n = 109,555** | **SMD** |
| --- | --- | --- | --- |
| **Test type**  CT  MRI  PICC  Ultrasound | 3543 (46.1)  707 (9.2)  547 (7.1)  2888 (37.6) | 57063 (52.1)  11463 (10.5)  7190 (6.6)  33839 (30.9) | 0.16 |
| **CT by anatomical region** Brain  Thorax  Abdomen/pelvis | 1337 (17.4)  1070 (13.9)  1222 (15.9) | 17840 (16.3)  20446 (18.7)  19995 (18.3) | 0.01  0.12  0.06 |
| **MRI by anatomical region**  Brain  Spine | 479 (6.2)  241 (3.1) | 8571 (7.8)  3163 (2.9) | 0.07  0.01 |
| **Ultrasound by anatomical region** Venous leg with Doppler  Abdomen  Extremity | 542 (7.1)  2168 (28.2)  181 (2.4) | 10512 (9.6)  21949 (20.0)  1498 (1.4) | 0.09  0.20  0.07 |
| **Time from admission to ordering test** Less than 1 day  1-2 days  2-4 days  4 or more days | 3630 (47.2)  700 (9.1)  916 (11.9)  2439 (31.7) | 51133 (46.7)  11920 (10.9)  13990 (12.8)  32512 (29.7) | 0.06 |
| **Median time from test ordering to completion, hours (IQR)** | 12.0 [2.4, 26.4] | 12.0 (2.4, 26.4) | 0.02 |
| **Median time spent waiting for tests, hours (IQR)** | 11.8 (3.0, 27.3) | 12.4 (3.1, 26.0) | 0.02 |
| **Proportion of total days spent waiting for tests, mean (SD)** | 0.1 (0.1) | 0.1 (0.1) | 0.02 |
| **Tests ordered in Emergency Department** | 2264 (29.5) | 29524 (26.9) | 0.06 |
| **Tests ordered in Intensive Care Unit** | 765 (10.0) | 7340 (6.7) | 0.12 |
| **Tests ordered during the weekend** | 2778 (36.1) | 38518 (35.2) | 0.02 |
| **Tests ordered overnight** | 1816 (23.6) | 25114 (22.9) | 0.03 |
| **Tests ordered while patient was bedspaced** | 1775 (23.1) | 25537 (23.3) | 0.00 |
| **GIM census, median (IQR)** | 92.0 (82.0, 104.0) | 95.0 (84.0, 111.0) | 0.23 |
| **Capacity ratio, median (IQR)** | 1.0 (0.9, 1.1) | 1.0 (0.9, 1.1) | 0.05 |
| **Income quintile**^a^ |  |  | 0.38 |
| Q1 | 2123 (27.6) | 28324 (21.7) |  |
| Q2 | 1031 (13.4) | 21819 (16.7) |  |
| Q3 | 937 (12.2) | 20694 (15.9) |  |
| Q4 | 806 (10.5) | 19943 (15.3) |  |
| Q5 | 939 (12.2) | 22863 (17.5) |  |
| Information Not Available | 1849 (24.1) | 16665 (12.8) |  |
| **Deprivation quintile**^b^ |  |  | 0.35 |
| Q1 | 1420 (18.5) | 28759 (22.1) |  |
| Q2 | 830 (10.8) | 18831 (14.5) |  |
| Q3 | 861 (11.2) | 18290 (14.0) |  |
| Q4 | 861 (11.2) | 21348 (16.4) |  |
| Q5 | 1855 (24.1) | 26023 (20.0) |  |
| Information Not Available | 1858 (24.2) | 17057 (13.1) |  |

SMD – standardized mean difference; CT – computed tomography; MRI – magnetic resonance imaging; PICC – peripherally-inserted central catheter; IQR – interquartile range. a – Q1 is lowest income and Q5 is highest income. b – Q1 is highest level of deprivation and Q5 is lowest level of deprivation.

**Supplemental table 1c. Tests ordered for patients with mood and anxiety disorders and without psychiatric comorbidity. Values are numbers (percentages) unless stated otherwise.**

| **Characteristic** | **Patients with mood and anxiety disorders**  **n = 12,470** | **Patients with no psychiatric comorbidity**  **n = 109,555** | **SMD** |
| --- | --- | --- | --- |
| **Test type**  CT  MRI  PICC  Ultrasound | 6450 (51.7)  1475 (11.8)  845 (6.8)  3700 (29.7) | 57063 (52.1)  11463 (10.5)  7190 (6.6)  33839 (30.9) | 0.05 |
| **CT by anatomical region** Brain  Thorax  Abdomen/pelvis | 2211 (17.7)  2137 (17.1)  2170 (17.4) | 17840 (16.3)  20446 (18.7)  19995 (18.3) | 0.02  0.02  0.01 |
| **MRI by anatomical region**  Brain  Spine | 1102 (8.8)  392 (3.1) | 8571 (7.8)  3163 (2.9) | 0.04 0.02 |
| **Ultrasound by anatomical region** Venous leg with Doppler  Abdomen  Extremity | 1049 (8.4)  2464 (19.8)  191 (1.5) | 10512 (9.6)  21949 (20.0)  1498 (1.4) | 0.03 0.01  0.01 |
| **Time from admission to ordering test** Less than 1 day  1-2 days  2-4 days  4 or more days | 5280 (42.3)  1194 (9.6)  1550 (12.4)  4446 (35.7) | 51133 (46.7)  11920 (10.9)  13990 (12.8)  32512 (29.7) | 0.11 |
| **Median time from test ordering to completion, hours (IQR)** | 14.4 (2.4, 28.8) | 12.0 (2.4, 26.4) | 0.05 |
| **Median time spent waiting for tests, hours (IQR)** | 13.9 (3.0, 28.0) | 12.4 (3.1, 26.0) | 0.06 |
| **Proportion of total days spent waiting for tests, mean (SD)** | 0.1 (0.1) | 0.1 (0.1) | 0.06 |
| **Tests ordered in Emergency Department** | 3224 (25.9) | 29524 (26.9) | 0.03 |
| **Tests ordered in Intensive Care Unit** | 786 (6.3) | 7340 (6.7) | 0.01 |
| **Tests ordered during the weekend** | 4250 (34.1) | 38518 (35.2) | 0.02 |
| **Tests ordered overnight** | 2801 (22.5) | 25114 (22.9) | 0.01 |
| **Tests ordered while patient was bedspaced** | 2875 (23.1) | 25537 (23.3) | 0.00 |
| **GIM census, median (IQR)** | 96.0 [85.0, 110.0] | 95.0 (84.0, 111.0) | 0.01 |
| **Capacity ratio, median (IQR)** | 1.0 (0.9, 1.1) | 1.0 (0.9, 1.1) | 0.00 |
| **Income quintile, median (IQR)** | 3.0 (1.0, 5.0) | 3.0 (2.0, 5.0) | 0.11 |
| **Income quintile**^a^ |  |  | 0.11 |
| Q1 | 3126 (25.1) | 27321 (21.8) |  |
| Q2 | 2122 (17.0) | 20728 (16.5) |  |
| Q3 | 2024 (16.2) | 19607 (15.6) |  |
| Q4 | 1846 (14.8) | 18903 (15.1) |  |
| Q5 | 1995 (16.0) | 21807 (17.4) |  |
| Information Not Available | 1357 (10.9) | 17157 (13.7) |  |
| **Deprivation quintile, median (IQR)** | 3.0 (2.0, 5.0] | 3.0 (2.0, 5.0) | 0.11 |
| **Deprivation quintile**^b^ |  |  | 0.11 |
| Q1 | 2920 (23.4) | 27259 (21.7) |  |
| Q2 | 1760 (14.1) | 17901 (14.3) |  |
| Q3 | 1577 (12.6) | 17574 (14.0) |  |
| Q4 | 2152 (17.3) | 20057 (16.0) |  |
| Q5 | 2689 (21.6) | 25189 (20.1) |  |
| Information Not Available | 1372 (11.0) | 17543 (14.0) |  |

SMD – standardized mean difference; CT – computed tomography; MRI – magnetic resonance imaging; PICC – peripherally-inserted central catheter; IQR – interquartile range. a – Q1 is lowest income and Q5 is highest income. b – Q1 is highest level of deprivation and Q5 is lowest level of deprivation.

**Supplemental figure 1. Creation of the cohort evaluated in the multivariable model.**

196,819 admissions to General Internal Medicine between
April 1, 2010 and December 31, 2019

No tests ordered during admission (n = 22,999 admissions)

Test(s) ordered before admission

(n = 96,258 admissions)

77,562 admissions (56,872 patients, 137,993 tests)
included in the multivariable model for time to test

MRP – most responsible provider, CPSO – College of Physicians and Surgeons of Ontario.
